# Evaluation of Quality Parameters in Canned Pork Enriched with 1% Freeze-Dried Cell-Free Supernatant of *Lacticaseibacillus paracasei* B1 and Reduced Sodium Nitrite Content

**DOI:** 10.3390/foods14173080

**Published:** 2025-09-01

**Authors:** Paulina Kęska, Miroslava Kačániová, Joanna Stadnik, Karolina Wójciak, Dorota Zielińska

**Affiliations:** 1 Department of Animal Food Technology, Faculty of Food Science and Biotechnology, University of Life Sciences in Lublin, Skromna 8, 20-704 Lublin, Poland; paulina.keska@up.lublin.pl (P.K.); karolina.wojciak@up.lublin.pl (K.W.); 2 Institute of Horticulture, Faculty of Horticulture and Landscape Engineering, Slovak University of Agriculture, Tr. A. Hlinku 2, 94976 Nitra, Slovakia; miroslava.kacaniova@uniag.sk; 3 School of Medical & Health Sciences, VIZJA University, Okopowa 59, 01-043 Warszawa, Poland; 4 Department of Food Gastronomy and Food Hygiene, Institute of Human Nutrition Sciences, Warsaw University of Life Sciences (SGGW), Nowoursynowska 159C St., (Building No. 32), 02-776 Warsaw, Poland; dorota_zielinska@sggw.edu.pl

**Keywords:** sodium nitrite alternatives, meat preservation, lactic acid bacteria

## Abstract

The search for natural alternatives to sodium nitrite in meat products is driven by concerns about consumer health and the need to maintain product quality and safety. In this study, the effect of sodium nitrite reduction on the quality parameters of canned pork meat with 1% lyophilized cell-free supernatant (CFS) from *L. paracasei* B1, during 30 days of storage, was assessed. Reduction of sodium nitrite content led to measurable changes in the color, texture, and oxidative stability of canned pork; however, the presence of 1% CFS helped preserve color, alleviated the negative impact on textural parameters, and limited lipid oxidation, thereby counteracting the typical consequences of nitrite reduction. Among the tested variants, S_75, containing 75% of the standard nitrite dose, showed the best overall balance between color retention, textural integrity, and oxidative stability. Samples without nitrite (S_0) exhibited a noticeable increase in lightness (L*) and decrease in redness (a*) over time, accompanied by a shift towards yellow-brown hues (b*, C*, *H*°). Importantly, the total color difference (Δ*E*) was least pronounced in the S_75 variant, with values of approximately 2.5 after 1 day and 2.7 after 30 days, which was markedly lower than in S_50 (Δ*E* ≈ 6.0 and 3.9) and S_0 (Δ*E* ≈ 7.9 and 8.5), thereby confirming superior color retention and overall stability during storage. Texture analysis showed that initial hardness and chewiness were higher in nitrite-free samples (S_0), suggesting that the complete omission of nitrite may negatively affect product structure. Nevertheless, all variants softened during storage, and samples with higher nitrite content, particularly S_75, retained better elasticity and cohesiveness. Lipid oxidation, expressed as TBARS values, progressed fastest in samples completely depleted of nitrite (S_0), increasing from 0.31 mg MDA/kg (day 1) to 1.35 mg MDA/kg (day 30), which confirms the antioxidant role of sodium nitrite. Interestingly, the presence of 1% CFS in the variants with reduced nitrite content partially mitigated this effect, as TBARS values in S_75 increased only from 0.29 to 0.46 mg MDA/kg, and, in S_50, from 0.45 to 0.66 mg MDA/kg, compared to the nitrite-free variant. This suggests that CFS may also have contributed to antioxidant protection. Fatty acid profiles remained relatively consistent across methods. Microbiological analysis revealed no significant differences between groups. These results demonstrate that partial nitrite reduction combined with CFS is effective, highlighting the potential of CFS as a promising functional additive in clean label meat preservation. Furthermore, reducing the sodium nitrite content in canned pork products may contribute to improved consumer health by reducing exposure to potentially harmful nitrosamine precursors.

## 1. Introduction

Sodium nitrate and nitrite, commonly used as preservatives in meat products, are associated with potential health risks due to their involvement in the formation of nitrosamines, compounds classified as probable carcinogens [1]. Reducing sodium nitrite levels in processed meat is therefore a priority in food science and public health to minimize exposure to these harmful substances. However, sodium nitrite also plays a key role in preserving meat quality by inhibiting lipid oxidation, stabilizing color, and controlling microbial growth [2,3]. The challenge is therefore to find strategies that can reduce health risks without negatively impacting the safety and sensory qualities of meat products.

Canned meats, including pork, are an important segment of the food processing market due to its long shelf life, microbiological safety, and convenient storage. Traditionally, their microbiological stability and color are maintained by the addition of nitrites (mainly sodium nitrite), the permissible limits of which are regulated by EU law. According to Commission Directive 2006/52/EC and Commission Regulation (EU) No 1129/2011, the maximum permissible concentration of nitrites in sterilized meat products is 100 mg/kg [4]. However, as noted by Ferysiuk and Wójciak [5], based on the Food Chain Evaluation Consortium report (2016), it was found that an average dose of 80 ppm may be sufficient to maintain the safety and sensory quality of canned meat products [5].

Growing consumer awareness and health concerns about nitrites, particularly their potential to form carcinogenic nitrosamines, have led to extensive research into natural, safe, and effective alternatives to preservatives. One of the most promising groups of natural antimicrobial compounds are metabolites produced by lactic acid bacteria (LAB). Both LAB bacteria and their metabolites have been recognized by the FDA as safe for use in food (GRAS: Generally Recognized as Safe). Among these alternatives, an innovative approach has emerged in the form of freeze-dried cell-free supernatant (CFS), which can compensate for the reduced preservative effect caused by the lower nitrite content. The bioactive compounds present in the CFS, including peptides, organic acids, and antioxidant metabolites, may provide natural antioxidant and antimicrobial activity, thereby enhancing product stability and safety without relying heavily on chemical preservatives [6]. This strategy also aligns with growing consumer demand for “clean label” products that feature natural ingredients and fewer synthetic additives.

CFS obtained from LAB cultures has been proven to effectively inhibit the growth of undesirable microorganisms in a wide range of food matrices, including fresh poultry and red meat, dairy products, bread, and various processed fruits and vegetables [7,8,9,10,11,12,13]. The use of CFS from lactic acid bacteria in meat products is a promising alternative to traditional chemical preservatives, offering benefits such as extending the shelf life of products, improving sensory properties, and meeting consumer expectations regarding the naturalness of ingredients. A study by Wang et al. [14] showed that the addition of 0.5% CFS from *Lactiplantibacillus plantarum* 90 (LCFS) to ground beef gel effectively inhibited the growth of microorganisms, extending the shelf life of the product to 8 days stored at 4 °C. Additionally, the use of LCFS did not negatively affect the pH, cooking, color, or the texture of the product. The mechanism of antimicrobial action of LCFS involves the interaction of organic acids and protein antibacterial substances, leading to damage to bacterial cell structures. In turn, Incilli et al. [15] assessed the effect of CFS from *Pediococcus acidilactici* on the shelf life of sausages. The use of 100% CFS combined with 1% chitosan as an edible coating extended the shelf life of the sausages to 28 days under refrigerated conditions, without significant changes in color and sensory properties. Additionally, inhibition of oxidative processes and antimicrobial activity against microorganisms responsible for product spoilage were observed. Moreover, in another study, the effect of CFS from different LAB strains on spoilage bacteria in vacuum-packed sausage-type cold cuts was assessed. CFS from *Lactobacillus acidophilus* ATCC 4356, *Lactiplantibacillus plantarum* CEC 17484, and *Pediococcus acidilactici* DSM 20284 showed over 88% effectiveness in inhibiting the growth of *Enterococcus mundtii*, *Latilactobacillus sakei*, *Latilactobacillus curvatus*, and *Weissella viridescens* [16]. Importantly, the activity of CFS metabolites was resistant to pasteurisation (85 °C for 10 min), which indicates their potential use as biopreservatives in the production of cold meats [16].

Additionally, to enhance antimicrobial activity and minimize the impact of the liquid form of CFS on the physicochemical properties of the product, it is concentrated by lyophilization. This process allows obtaining a stable powder with a high concentration of metabolites, which facilitates its introduction into the food structure and increases the technological potential of its use. The literature has shown that lyophilized CFS with *Lactobacillus salivarius* (Ls-BU2), at a concentration of 35 mg/g, effectively inhibited the growth of psychrotrophic spoilage bacteria in ground beef stored at 4 °C. Additionally, an improvement in the sensory properties of the product was observed, which suggests the potential of CFS as a food additive to control pathogens and extend the shelf life of ground meat [8]. However, most research to date has focused on fresh, chilled, or fermented products, and information on the use of CFS in heat-sterilized products, such as canned meats, is limited. This highlights the importance of exploring the use of CFS in this sector to develop safe and natural preservation strategies.

From a health perspective, reducing sodium nitrite while maintaining product quality through natural bacterial metabolites may reduce the risk of nitrosamine formation and related chronic diseases, including some cancers. Furthermore, reducing chemical additives contributes to lowering the total intake of sodium and related compounds, which is beneficial in the treatment of hypertension and cardiovascular disease [17]. Therefore, to support the development of safer and healthier meat products and to address both regulatory guidelines and consumer health concerns, this study was conducted. It was hypothesized that partial sodium nitrite reduction, combined with the addition of 1% CFS as a natural bacterial metabolite, would maintain the antioxidant protection and overall quality of canned pork products while reducing the potential health risks associated with nitrosamines derived from nitrites. The results of this work could provide a basis for the development of new, natural biopreservation strategies in heat-sterilized products, benefiting consumer health.

## 2. Materials and Methods

### 2.1. Obtaining Lyophilized Cell Free Supernatant (CFS)

In this study, the strain of lactic acid bacteria *Lacticaseibacillus paracasei* B1 (GenBank accession no: CP161807 and CP161808) was obtained from the internal microorganism collection of the Department of Food Gastronomy and Food Hygiene at the Institute of Human Nutrition Sciences, Warsaw University of Life Sciences (Poland), where was storage as deeply frozen state at −80 °C with a 20% addition of glycerol in De Man Rogosa and Sharpe broth (MRS, Oxoid LTD, Hampshire, UK). Prior to experimental use, bacterial cultures were revived by incubation in fresh MRS broth at 37 °C for 24 h. Next, the stain was incubated for 48 h in food liquid medium prepared according to Jeong et al. [18] at temp. 37 °C under aerobic conditions to restore bacterial cultures, to achieve approx. 10^9^ CFU/mL count. The medium contained lactose (Sigma-Aldrich, St. Louis, MO, USA) (2.5%, *w*/*v*) as carbon sources, yeast extract (A&A Biotechnology, Gdańsk, Poland), and whey protein concentrate (WPC) (Mlekowita, Wysokie Mazowieckie, Poland) in a 4:1 ratio as nitrogen source (2.5% *w*/*v*). Potassium citrate (Biomus, Lublin, Poland) was used as a pH buffer (3%, *w*/*v*), and ascorbic acid (Biomus, Lublin, Poland) was used as an antioxidant (0.05%; *w*/*v*). The bacterial cultures were subjected to the following treatments: step 1: heat treatment (100 °C; 10 min); step 2: centrifugation (8000× *g*; 10 min). Next, supernatants were collected, and cell-free supernatant (CFS) was frozen and lyophilized for 72 h using a freeze dryer (Free Zone 12 lyophilizer, Labconco Corporation, Kansas City, MO, USA) at −80 °C and 0.04 mbar. The lyophilizate was stored at room temperature until analysis.

### 2.2. Meat Product Preparation

The meat used in the study came from Polish Large White pigs (*Sus scrofa*) with a live weight of 120–130 kg. The raw material was obtained from half-carcasses chilled at 7 °C 48 h after slaughter. The raw material consisted of 850 g/kg of pork ham (*Musculus biceps femoris*) and 150 g/kg of pork backfat. The ham muscles were initially minced with a knife and then ground in a KU2-3EK universal meat grinder (MESKO-AGD, Skarżysko-Kamienna, Poland) with an 8 mm diameter disc. The ground meat was then mixed with ground backfat. The resulting meat stuffing was divided into four parts to which different amounts of sodium nitrite were added: S_100, with 100 mg/kg addition; S_75, with 75 mg/kg addition; S_50, with 50 mg/kg addition; and S_0, with no nitrite addition. While mixing the meat stuffing, a curing mixture containing sodium nitrite or sodium chloride was added at a level of 28 g/kg of meat. Additionally, water (S_100) or CFS lyophilizate dissolved in water (1%; S_75, S_50, S_0) was added at a level of 50 mL/kg. After mixing (KU2-3E, Mesko-AGD, Skarżysko-Kamienna, Poland; 4–5 min), the meat stuffing was transferred to aluminum, enameled cans with lids (intended for contact with food; Ø 99 mm, height: 47 mm; capacity: 300 mL) until they were almost full. The cans were tightly closed and thermally treated in a sterilizer (vertical steam type, TYP-AS2, Polsonic, Warsaw, Poland). The experimental samples were heated at 121 °C, assuming that their degree of heating was achieved as measured with the sterilization value (F ≈ 4 min). After sterilization, the cans were cooled in water and stored at 4 °C in the refrigerator. The canned meats were analyzed immediately after production (day 1), and after 30 days of storage.

### 2.3. Physicochemical (pH, a_w_, ORP) Properties

Samples for total acidity (pH) measurements were obtained after cutting a slice from the center of the sausage. The ground meat sample was homogenized (T25 Basic ULTRA-TURRAX; IKA, Staufen, Germany) for 1 min with distilled water in a ratio of 1:10. The pH was measured in the homogenate using a temperature-compensated digital pH meter (CPC-501; Elmetron, Zabrze, Poland) equipped with combined glass pH electrode (ERH-111; Hydromet, Gliwice, Poland). The pH meter was standardized with buffer solutions at pH 4.0, 7.0, and 9.0, prior to pH determination.

The oxidation-reduction potential was determined in meat homogenate prepared as above by a combined platinum electrode, type ERPt-13, using a digital pH conductometer CPC-501 (Elmetron, Zabrze, Poland).

Water activity (*a_w_*) was measured using a laboratory apparatus (Novasina AG, Lachen, Switzerland) in a temperature-controlled measurement chamber. A sample (5 g) of the product was placed in the vessel of the device, and the analysis was performed automatically at 20 °C. The Novasina humidity standards, prepared using saturated salt solutions, were employed for calibration.

### 2.4. Antioxidant Activity of Meat Product Extract

To obtain the sausage extract, 10 g of the product was homogenized for 1 min on ice with 90 mL of distilled water. The homogenate was kept under refrigeration for 60 min, then centrifuged and the supernatant collected. Before assessing the antioxidant potential, the extract was purified using a sterile filter.

#### 2.4.1. Radical Scavenging Activity

Scavenging activity against the 2,2′-azino-bis (3-ethylbenzothiazoline-6-sulfonic acid (ABTS) (Glentham Life Sciences, Corsham, UK)) radical cation was tested according to Re et al. [19]. This test is based on the decolorization of the green chromophore of the ABTS radical cation (ABTS^•+^). The radical solution was prepared by reacting ABTS with potassium persulfate (Sigma-Aldrich, St. Louis, MO, USA), then diluted in distilled water to a final concentration of 2.45 mM, then incubated in the dark for 16 h to allow for complete radical formation. Before use, the solution was diluted to an absorbance of approximately 0.70–0.72. A 180 µL sample of ABTS was mixed with 20 µL of the CFS in a 96-well microtiter plate. After shaking the mixture, it was transferred to a dark chamber and left for 5 min at ambient temperature. Subsequently, the measurement of the absorbance at 734 (ABTS) was applied in order to calculate the scavenging activity using the given equation:

(1) ABTS activity [%] = (A_Control_ − A_Sample_)/A_Control_) × 100

#### 2.4.2. Determination of Reducing Power

The RP of the samples was determined by the method described by Oyaizu [20]. For this purpose, 500 μL of sodium phosphate buffer (pH 6.6) and 500 μL of 1% potassium hexacyanoferrate(II) solution (K_4_[Fe(CN)_6_]) (Chempur, Piekary Śląskie, Poland) were added to 500 μL of extract. The prepared mixture was incubated for 20 min in a water bath at 50 °C. Then, 500 μL of 10% trichloroacetic acid (POCH, Gliwice, Poland) was added and the sample was centrifuged for 15 min at 5000× *g*. After centrifugation, 1 mL of supernatant was collected, to which 1 mL of deionized water and 200 μL of 0.1% iron(III) chloride (FeCl_3_) (Sigma-Aldrich, St. Louis, MO, USA) were added. The samples were left for 10 min, then the absorbance was measured at 700 nm using a Hitachi U-5100 UV-Vis spectrophotometer (Hitachi, Tokyo, Japan).

### 2.5. Thiobarbituric Acid Reactive Substance (TBARS) Status

Lipid oxidation was determined by assessing TBARS values according to the Pikul et al. [21] method. The color intensity produced in the reaction of malondialdehyde with 2-thiobarbituric acid was measured using the Nicolet Evolution 300 spectrophotometer (Thermo Electron Corp., Waltham, MA, USA) at a wavelength of 532 nm. TBARS values were expressed in mg malondialdehyde (MDA) per 1 kg of meat product.

### 2.6. Fatty Acid (FAA) Status

For fatty acid analysis, the method of Folch et al. [22] was used for lipid extraction from samples of sausages. The composition of fatty acids was determined by gas chromatography after concentrating version of fats with fatty acid methyl esters (FAME) according to the AOAC method [23]. The analysis was performed using a Varian 450-GC gas chromatograph (Varian, Inc., Santa Clara, CA, USA) equipped with a split/splitless injector, a flame ionization detector (FID), and a 30-m fused silica capillary column (internal diameter 0.32 mm). Helium was used as the carrier gas. The injected sample volume was 1 µL, and the injection was performed in split mode with a split ratio of 1:50. The injector and detector temperatures were set to 250 °C and 300 °C, respectively. After injection, the column oven was programmed as follows: an initial temperature of 200 °C was held for 10 min, followed by a ramp of 3 °C min^−1^ until reaching 240 °C, which was then maintained for an additional 4 min. Fatty acid contents were quantified based on chromatograms and internal standard calibration. The reference standard used was Supelco 37 Component FAME Mix (Sigma-Aldrich, St. Louis, MO, USA). The following fatty acids were analyzed: C6:0 (caproic acid); C8:0 (caprylic acid); C10:0 (capric acid); C12:0 (lauric acid); C14:0 (myristic acid); C14:1n5 (oleomyristic acid); C15:0 (pentadecanoic acid); C16:0 (palmitic acid); C16:1n7 (palmitoleic acid); C17:0 (margaric acid); C17:1n7 (cis-9-heptadecenoic acid); C18:0 (stearic acid); C18:1n9C (oleic acid); C18:1n9t (elaidic acid); C18:2n6c (linoleic acid); C18:2n6t (linolelaidic acid); C18:3n3 (alpha) (alpha-linolenic acid); C20:0 (arachidonic acid); C20:1n15 (cis-5-eicosenoic acid); C20:1n9 (cis-11-eicosenoic acid); C20:2n6 (cis-11,14-eicosadienoic acid); C21:0 (heneicosanoic acid); C20:4n6 (arachidonic acid); C20:5n3 (cis-5,8,11,14,17-eicosapentaenoic acid); C22:0 (behenic acid); C22:1n9 (erucic acid); C22:6n3 (cis-13,16-docosadienoic acid); and C24:1n9 (nervonic acid). The final results were presented as follows: saturated fatty acids (SFA); monounsaturated fatty acids (MUFA); polyunsaturated fatty acids (PUFA); OMEGA 3 as sum of fatty acid: C18:3n3 (alpha), C20:3n3, C20:5n3, C22:6n3; OMEGA 6 as sum of fatty acid: C18:2n6c, C18:3n6 (gamma), C20:2n6, C20:3n6, C20:4N6, C22:2n6; OMEGA 9 as sum of fatty acid: C18:1n9c, C20:1n9, C22:1n9, C24:1n9. All values were expressed as percentages [%] of the total identified fatty acid pool.

### 2.7. Instrumental Color (L*a*b*)

The samples were assessed for instrumental color using a laboratory spectrophotometer (Colour^®^ Premiere 8200; X-Rite Inc., Grand Rapids, MI, USA). Color measurement followed the Commission Internationale de l’Eclairage (CIE) (1978) [24] color convention, outputting L* (lightness), a* (redness), and b* (yellowness). Also, C* (chroma) and *H*° (hue angle) were calculated using the standard equations:

(2) $$C^{*}={\left[{\left(a^{*}\right)}^{2}+{\left(b^{*}\right)}^{2}\right]}^{\frac{1}{2}}$$

(3) $$H{}^{\circ}=arctan\frac{b^{*}}{a^{*}}$$

The measurement was conducted using the following parameters: D65 illumination (daylight, 6500 K), standard observer 10°. The instrumental conditions were a 12 mm diameter area aperture. The measurement was carried out in the range of 360 to 740 nm. Before color analysis, the samples were removed from the refrigerator for 20 min, then cut crosswise. The color measurement was performed on a 5 cm slice of meat bar, immediately after cutting. The total color difference (Δ*E*) between the pattern and the sample was calculated according to the following equation:

(4) $$∆E=\sqrt{{{(L}_{pattern}-L_{sample})}^{2}+{{(a}_{pattern}-a_{sample})}^{2}+{{(b}_{pattern}-b_{sample})}^{2}}$$

Total color difference (Δ*E*) between the pattern (control sample, S_100) and the remaining samples (i.e., S_75, S_50, and S_0) was calculated.

### 2.8. Texture Profile Analysis (TPA)

Texture profile analysis (TPA) was performed using a texture analyzer (TA-XT2i, Stable Micro Systems Ltd., Surrey, UK). Before analysis, the samples were allowed to reach room temperature for 30 min, after which six cylindrical cores (25 mm length × 20 mm diameter) were cut for each variant. The samples were compressed twice between two parallel plates to 50% of their original height, with a time interval between the two compression cycles of 5 s, at a crosshead speed of 2 mm/s. The TA-25 with a 2-inch diameter stainless steel probe was used. TPA recorded the following attributes: hardness [g], springiness [%], cohesion [-], gumminess [-], and chewiness [-].

### 2.9. Microbiological Analysis

Five grams of the sample were mixed with 45 mL of a sterile saline solution (0.1%) in an Erlenmeyer flask. The mixture was then subjected to an incubation period of 30 min within a shaking incubator (GFL 3031, Burgwedel, Germany). A range of bacteria were the subject of investigation as part of the evaluation. Coliforms were identified using Violet Red Bile Lactose Agar (VRBL, Oxoid, Basingstoke, UK) during a 24–48 h incubation period at 37 °C. Plate Count Agar (PCA; Oxoid, Basingstoke, UK) was utilized to calculate total viable counts (TVC), with the cultures being maintained at 30 °C for a period of 48–72 h. De Man Rogosa and Sharpe Agar (MRS; Oxoid, Basingstoke, UK) was utilized for the cultivation of lactic acid bacteria for a period of 48–72 h at a temperature of 37 °C. Each analysis and test was conducted in three separate replications. Furthermore, as part of a rapid reinoculation process, eight colonies per Petri dish were cultivated on Tryptone Soya Agar (TSA; Oxoid, Basingstoke, UK) at 30 °C [25].

#### 2.9.1. Microorganism Identification Using Mass Spectrometry

Microorganisms isolated from canned pork meat samples were identified using the MALDI-TOF (Matrix-Assisted Laser Desorption/Ionization Time of Flight) MS Biotyper system (Bruker Daltonics, Bremen, Germany), with the aid of reference libraries. For matrix preparation, a stock solution was first prepared and subsequently converted into an organic solution. Eight representative colonies from Petri plates were selected, and the biological material was transferred into Eppendorf tubes containing 300 µL of distilled water. The suspension was vortexed and centrifuged for 2 min at 10,000× *g* using a ROTOFIX 32A centrifuge (Ites, Vranov, Slovakia). Afterwards, 900 µL of ethanol was added. The supernatant was removed, and the pellet was air-dried at room temperature (20 °C). The dried pellet was then treated with 30 µL of 70% formic acid and 30 µL of acetonitrile. For identification, score values were interpreted as follows: <1.700, unreliable identification; 1.700–1.999, probable genus identification; 2.000–2.299, secure genus identification with possible species identification; 2.300–3.000, highly probable species identification.

#### 2.9.2. MALDI-TOF Biotyper Matrix Solution

After beginning as a stock solution, the MALDI-TOF Matrix Solution experienced an organic material-based change. The standard solution consisted of 2.5% trifluoroacetic acid, 47.5% water, and 50% acetonitrile. To make 1 mL of the stock solution, combine 475 µL of filtered water, 25 µL of pure 10% trifluoroacetic acid, and 500 µL of pure 100% acetonitrile. The organic solvent was combined with the “HCCA matrix portioned” in a 250 mL Eppendorf flask. Aloqence in Vrable, Slovakia, provided all matrix components. Subsequently, the samples were identified as previously reported in Kačániová et al. [25].

### 2.10. Statistical Analysis

The data were analyzed using factorial analysis of variance (ANOVA) in Statistica 13 (StatSoft Inc., Tulsa, OK, USA) software. Differences in mean values between groups were assessed using Tukey’s multiple comparison test, with significance set at *p* < 0.05. The experiment was conducted in two independent series, with at least five replicates for each treatment variant.

## 3. Results

Table 1 presents the results of the evaluation of the physicochemical properties of preserved pork. After 30 days of storage of the pork canned meat, a decrease in the water activity value (a*_w_*) (*p* < 0.05) and an increase in the pH value and oxidation-reduction potential (ORP) (*p* < 0.05) were observed in all the research variants. On the first day of the analysis (day 1), no statistically significant differences (*p* > 0.05) were found in the a*_w_* value between the individual variants, while, after 30 days of storage, water activity decreased significantly, reaching the lowest value in the variant without the addition of sodium nitrite (S_0). In relation to the pH value, a significant effect of the sodium nitrite dose on this parameter was observed. On the day of production, the highest pH value was recorded in the S_100 variant (with the addition of 100 mg of sodium nitrite/kg of stuffing, *p* < 0.05), then in the subsequent variants with a decreasing dose of nitrite (S_100 > S_75 > S_50 > S_0). After 30 days of storage, the pH value was significantly lower in the variant without added nitrite, while no significant differences were found between variants S_100, S_75, and S_50. The value of oxidation-reduction potential (ORP) after the storage period also depended on the applied dose of sodium nitrite. The lowest ORP value was recorded in variant S_100, while the highest was recorded in the control sample without added sodium nitrite (S_0) (*p* < 0.05).

**Figure 1:**
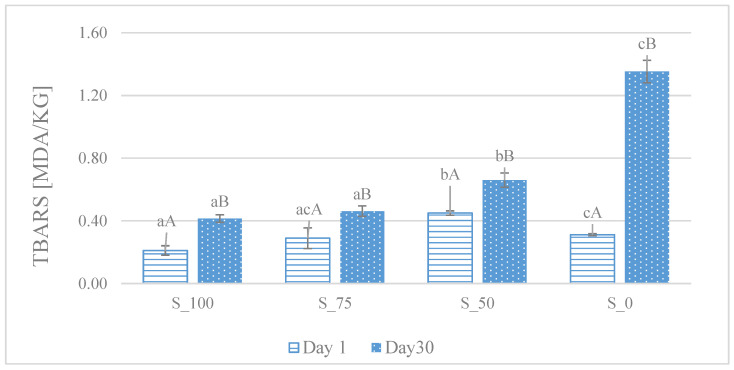
Content of secondary fat oxidation products defined as thiobarbituric acid reactive substances (TBARS) in canned pork meat with 1% CFS and reduced nitrite amount after production (day 1) and storage (4 °C; day 30). [S_100—variant with a sodium nitrite dose of 100 mg kg^−1^; S_75—variant with a sodium nitrite dose of 75 mg kg^−1^; S_50—variant with a sodium nitrite dose of 50 mg kg^−1^; S_0—nitrite-free variant. ^a–c^—different lowercase letters indicate significant differences between variants within the same day; ^A,B^—different uppercase letters indicate significant differences between days within the same variant (*p* < 0.05)].

Figure 1 shows the TBARS index values in pork canned samples analyzed in different variants of sodium nitrite addition. In all tested variants, an increase in TBARS level was observed after 30 days of storage, which indicates increasing lipid oxidation processes over time. The lowest TBARS values, both on the day of production (day 1) and after the storage period (day 30), were recorded in samples of variant S_100 (100 mg sodium nitrite kg^−1^ of stuffing), which confirms the antioxidant properties of the full dose of sodium nitrite. In turn, the highest TBARS values after 30 days were found in the variant without the addition of nitrite (S_0), which indicates a much higher susceptibility of the product to oxidative processes. Importantly, the addition of 1% CFS appeared to mitigate lipid oxidation in variants with reduced nitrite content (S_75 and S_50), as reflected by lower TBARS values compared to the nitrite-free variant (S_0). In the analyzed period (30 days), no statistically significant differences (*p* > 0.05) were found in the level of secondary fat oxidation products, marked with the TBARS index, between the control variant (S_100) and the variant with a 25% reduced sodium nitrite content (S_75), highlighting that CFS contributes to antioxidant protection and may allow the partial reduction of sodium nitrite without compromising product stability.

Analysis of the fatty acid profile of pork canned meats is presented in Table 2.

In terms of saturated fatty acids (SFA), the highest content was recorded in variant S_0 (48.50 ± 0.287%), while the lowest was recorded in S_75 (46.35 ± 0.287%). The main representative of this group was palmitic acid (C16:0), the share of which ranged from 28.53% (S_75) to 29.56% (S_100). Among monounsaturated fatty acids (MUFA), oleic acid (C18:1n9c + C18:1n9t) dominated, constituting 36.64% (S_50) to 40.40% (S_75) of the total lipid profile. Total MUFA content ranged from 43.62% to 44.93%, showing no significant differences between variants (*p* > 0.05). Polyunsaturated fatty acids (PUFA) were present in lower concentrations—their sum was the highest in variant S_75 (2.97 ± 0.033%) and the lowest in S_100 (2.08 ± 0.035%). The specific levels of omega-6 acids were also highest in S_75 (2.83 ± 0.040%) and lowest in S_100 (2.10 ± 0.042%). The content of omega-3 acids remained at the level of 0.07–0.14%, with the highest share recorded in variant S_75. The total content of omega-9 acids did not differ significantly between samples and was around 40%, with the highest value in S_75 (40.88 ± 0.024%) and the lowest in S_100 (39.68 ± 0.092%).

In terms of ABTS activity [%], significant differences were observed between variants at both time points (*p* < 0.05) (Table 3).

On day 1, the highest activity was shown by variant S_100, followed by S_75, with differences between them not being statistically significant, despite the 25% reduced dose of sodium nitrite in variant S_75. This may indicate a significant effect of the CFS additive content on the ability to scavenge radicals. Variants with a lower share (S_50 and S_0) showed significantly lower activity. After 30 days of storage, ABTS values decreased for variants S_100 and S_75, while in samples S_50 and S_0 there was a slight increase in activity, however, the antiradical activity was directly proportional to the dose of nitrites (S_100 > S_75 > S_50 > S_0) at that time. In the case of reducing power, an opposite trend was observed. On day 1, the lowest value was shown by variant S_100 (*p* < 0.05), and the highest by S_50 and S_75 (*p* > 0.05). After 30 days, all variants showed a significant increase in reducing power. The value in sample S_100 increased to 1.66 ± 0.02, and in the remaining variants it remained at a similar level (1.68–1.71), but the differences between them were not significant (*p* > 0.05). The exception was the nitrite-free variant (S_0), for which the lowest value of reducing salt was recorded (day 30).

**Figure 2:**
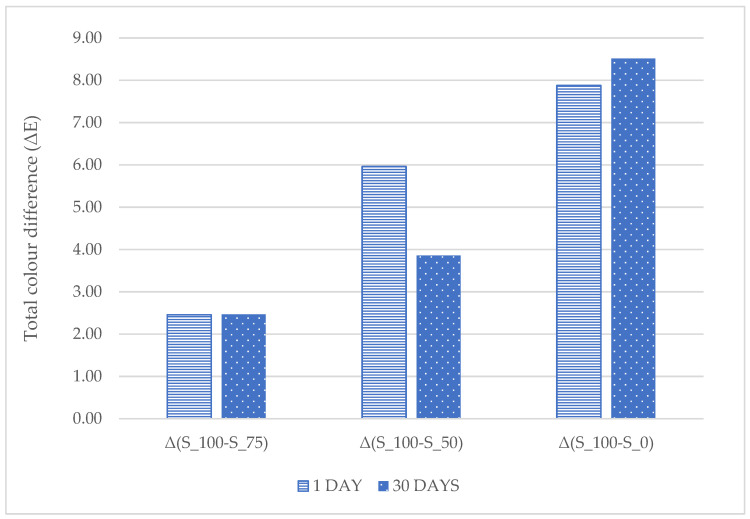
Total color difference (Δ*E*) between canned pork meats after production (day 1) and 30 days of storage (4 °C) [S_100—variant with a sodium nitrite dose of 100 mg kg^−1^; S_75—variant with a sodium nitrite dose of 75 mg kg^−1^; S_50—variant with a sodium nitrite dose of 50 mg kg^−1^; S_0—nitrite-free variant.

Effect of different doses of sodium nitrite (100%, 75%, 50% and 0%) on colorimetric parameters (L*, a*, b*, C*, *H*°) of pork canned meat with 1% CFS (Table 4) and total color difference (Δ*E*) compared to the control sample (S_100) (Figure 2) were investigated.

In terms of the L* parameter (lightness), no significant changes were observed during storage, except for variant S_0, in which a statistically significant increase in lightness was noted after 30 days (*p* < 0.05). A similar trend was observed for the a* parameter (redness), where, after 30 days of storage, no significant changes were observed in the a* values in the variants with added nitrite, while, in sample S_0, a significant decrease in this parameter was noted (*p* > 0.05). The b* values (yellowness) showed an upward trend with the decrease in the sodium nitrite dose, regardless of storage time. The highest b* values were recorded in variant S_0, which suggests a shift in the meat color towards yellow-brown tones. Changes in this parameter during storage were not statistically significant, which indicates the stability of the yellowness parameter in pork canned meats. Chromaticity (C*), which is an indicator of color saturation, was highest in variant S_0 and lowest in S_100, regardless of storage time (*p* < 0.05). A significant increase in the C* value was found only in variant S_75 (*p* < 0.05) between day 1 and day 30 of storage. The hue angle (*H*°) values were highest in sample S_0 (*p* < 0.05), which corresponds to a shift in the meat color towards yellow-brown. This is consistent with the simultaneous decrease in redness (a*) and increase in yellowness (b*) observed for this variant. The *H*° value was stable during the storage period, except for variant S_0, in which the value of this parameter decreased (*p* < 0.05) on day 30 of the analysis.

**Figure 3:**
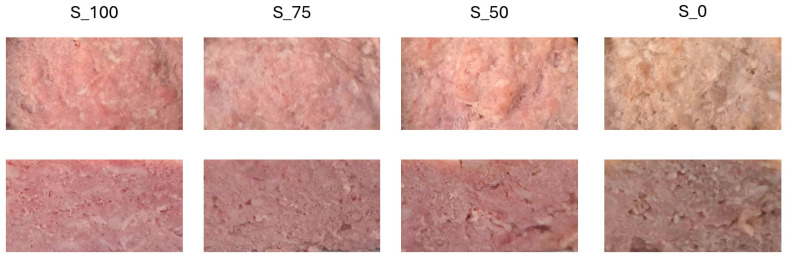
Canned pork meats with 1% CFS and reduced nitrite amount after production (day 1) (above: top view; below: cross-sectional view) [S_100—variant with a sodium nitrite dose of 100 mg kg^−1^; S_75—variant with a sodium nitrite dose of 75 mg kg^−1^; S_50—variant with a sodium nitrite dose of 50 mg kg^−1^; S_0—nitrite-free variant].

The results of the analysis of the total color difference (Δ*E*) between the control sample (S_100) and the variants with reduced nitrite content indicate that the smallest color difference occurs for sample S_75, both on day 1 and after 30 days of storage. Maintaining a low level of Δ*E* in this variant confirms that reducing the sodium nitrite dose by 25% does not significantly affect the color perception of the product. Sample S_50 showed significantly higher Δ*E* values compared to S_100 on the first day (Δ*E* ≈ 6), which decreased after 30 days of storage (Δ*E* ≈ 3.9) (Figure 3).

This may indicate that the optical properties of the samples are levelling out during storage, which is potentially related to oxidative changes in meat pigments. The highest Δ*E* values were recorded in sample S_0, both on day 1 and after 30 days of storage. This difference exceeded 8 and increased slightly over time, which indicates a lack of meat color stabilization in the complete absence of sodium nitrite (Figure 2 and Figure 3).

As presented in Table 5, the hardness of preserved pork showed a statistically significant increase (*p* < 0.05) with decreasing sodium nitrite content on day 1 of storage, with the values increasing successively in samples S_100, S_75, S_50, and S_0.

After 30 days of storage, hardness decreased significantly in all analyzed variants (*p* < 0.05), with the highest values maintained in the sample without sodium nitrite (S_0). In contrast, cohesion increased significantly over the same period in all variants (*p* < 0.05), regardless of sodium nitrite content. On day 1, the lowest cohesion was observed in S_0 (0.270), and the highest was observed in S_50 (0.351); after 30 days, differences between the groups were not statistically significant (*p* > 0.05), suggesting a leveling of the gel structure during storage. Springiness also increased significantly in all variants (*p* < 0.05), with the highest values observed in samples with higher sodium nitrite levels (S_75 and S_100), indicating the potential role of sodium nitrite in maintaining product elasticity. Gumminess increased both with storage time and with decreasing sodium nitrite content, with higher values in low-nitrite samples after 30 days. Chewiness differed significantly between variants on day 1 (*p* < 0.05): it was lowest in S_100 and highest in S_0. After 30 days, chewiness increased in all variants (*p* < 0.05), with the highest values in S_0 and S_50, confirming the effect of reduced sodium nitrite on this parameter.

Microbiological analysis, including determination of the number of coliform bacteria, total aerobic microorganisms (TVC: Total Viable Count), and lactic acid bacteria, showed no statistically significant differences between samples containing a full dose of sodium nitrite (S_100) and variants in which its amount was gradually reduced (S_75, S_50, S_0) (*p* > 0.05) (Table 6). The average number of coliform bacteria ranged from 1.56 to 1.68 log CFU g^−1^, with no significant effect of sodium nitrite reduction on this parameter. Similarly, the total number of aerobic microorganisms ranged from 1.58 to 1.76 log CFU g^−1^, and lactic acid bacteria ranged from 1.52 to 1.68 log CFU g^−1^, which also did not show any significant differences between the variants tested.

**Figure 4:**
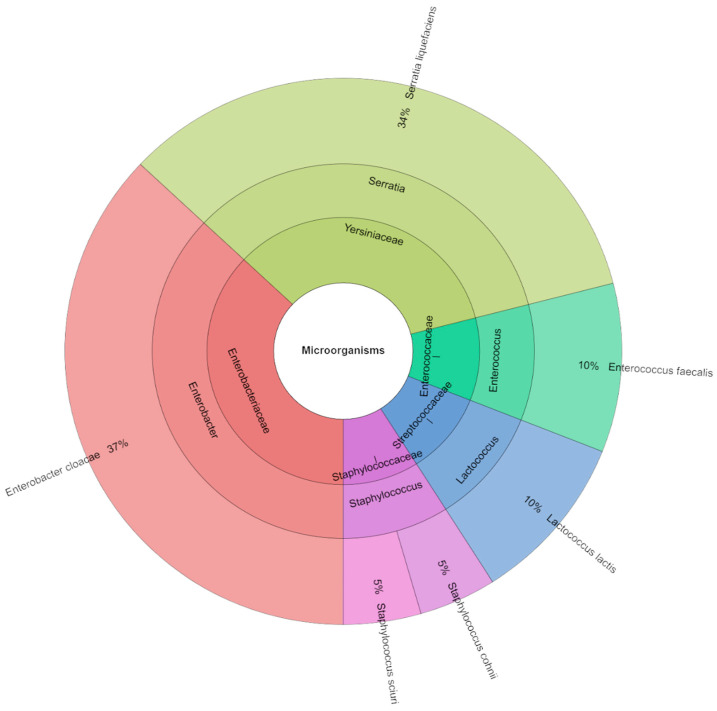
Microbiological profiles in nitrite-free variant (S_0).

Figure 4 lists the families, genera, and species of bacteria isolated from canned pork in the samples of variant-free nitrite. In total, 103 isolates were identified with mass spectrometry. The most frequently isolated families were *Enterobacteriaceae* and *Yersiniaceae*. The most identified species were *Enterobacter cloacae* (37%) and *Serratia liquefaciens* (34%).

**Figure 5:**
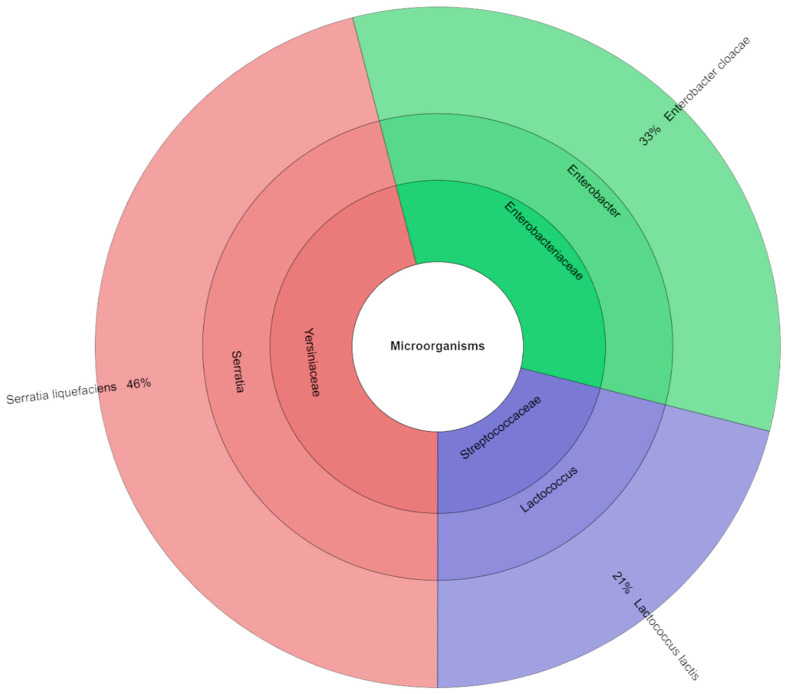
Microbiological profiles in variant with a sodium nitrite dose of 50 mg kg^−1^ (S_50).

As presented in Figure 5, the family, genera, and species of bacteria isolated from canned pork in the samples of the variant with a sodium nitrite dose of 50 mg kg^−1^ are listed. The utilization of mass spectrometry enabled the identification of a total of 94 isolates. It was observed that the most prevalent families isolated were *Enterobacteriaceae* and *Yersiniaceae*. Following a thorough analysis, the most prevalent species identified were *Serratia liquefaciens* (46%) and *Enterobacter cloacae* (33%).

**Figure 6:**
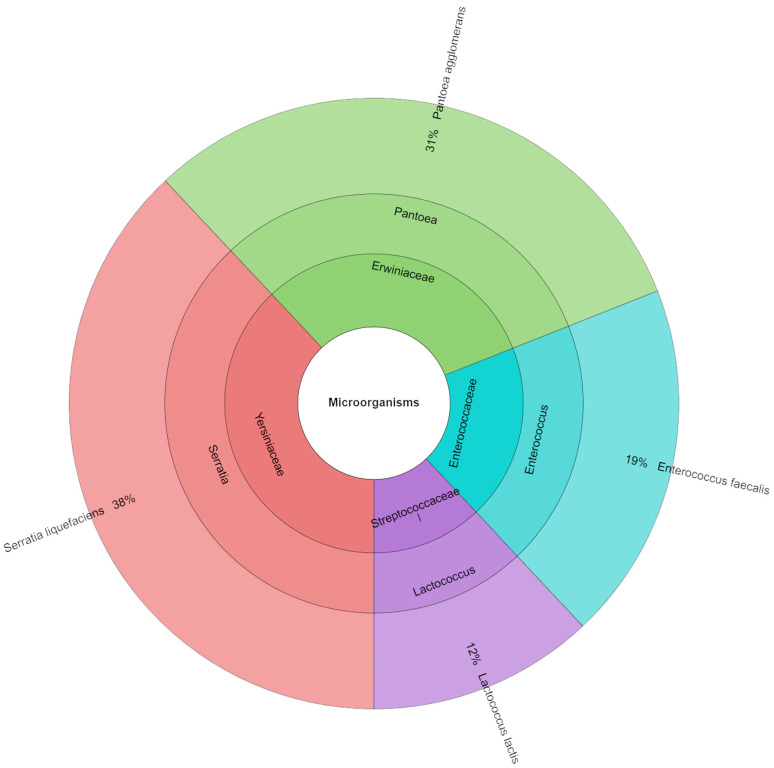
Microbiological profiles in variant with a sodium nitrite dose of 75 mg kg^−1^ (S_75).

Figure 6 provides a categorized overview of the bacterial isolates, encompassing families, genera, and species, isolated from the samples of canned pork that received a sodium nitrite dosage of 75 mg kg^−1^. In total, 80 isolates were successfully identified using mass spectrometry analysis. The most prevalent families isolated were *Enterobacteriaceae* and *Yersiniaceae*. The most prevalent species identified in the study were *Serratia liquefaciens* (38%) and *Pantoea agglomerans* (31%).

**Figure 7:**
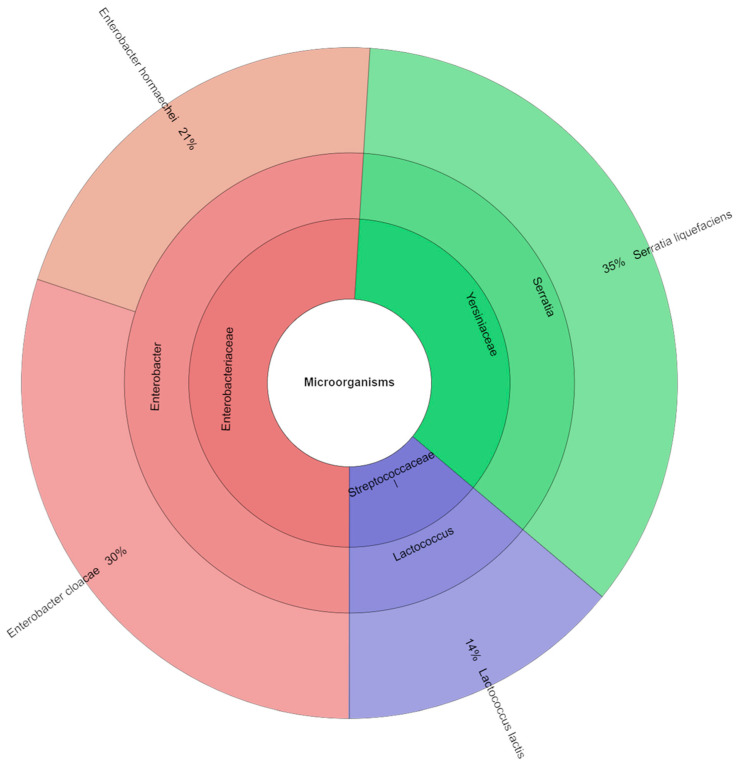
Microbiological profiles in variant with a sodium nitrite dose of 100 mg kg^−1^ [S_100].

Figure 7 presents a list of the bacteria isolated from canned pork in the variant with a sodium nitrite dose of 100 mg kg^−1^, categorized by family, genera, and species. A total of 72 isolates were identified through a combination of mass spectrometry techniques. The most frequently isolated families were identified as *Enterobacteriaceae* and *Yersiniaceae*. The most prevalent species identified in this study were *Enterobacter cloacae*, accounting for 30% of the total, and *Serratia liquefaciens*, accounting for 35%.

## 4. Discussion

It is claimed that it is impossible to eliminate sodium nitrite from the production of preserved meat [5]. However, it is possible to limit the use of this preservative, but by compensating for the loss of sodium nitrite with the addition of other factors, for example, lyophilized willow herb extract [26]. However, a critical approach to the use of plants in meat products is presented by Estévez [27], who suggests that, in addition to many desirable compounds (mainly antioxidants), various antinutritional compounds are introduced to meat products along with the plant additive. Therefore, in this study, the possibility of reducing the addition of sodium nitrite was assessed, while using a 1% addition of lyophilized CFS. The bacterial origin of CFS eliminates the hazards associated with the use of plant additives. From a health perspective, reducing sodium nitrite is crucial due to concerns about the potential formation of carcinogenic N-nitrosamines in the human body. Using bacterial CFS as a natural additive can help maintain product quality while reducing the health risks associated with synthetic preservatives.

### 4.1. Physiochemical Parameters

In the present work, after 30 days of storage of preserved pork, a decrease in a*_w_* was observed in all variants. No significant differences in a*_w_* were found between samples with different doses of sodium nitrite, although the lower a*_w_* in the nitrite-free sample (S_0) may indicate a greater extent of chemical reactions, including the recombination of macromolecules (proteins, fats) that bind free water [28]. An increase in both pH and oxidation-reduction potential (ORP) was also observed. The rise in pH may result from the decomposition of nitrogen compounds, such as peptides and amino acids, releasing alkaline products (e.g., ammonia). Further, the processing process, i.e., the use of high temperatures during sterilization, contributes to the denaturation of proteins, which affects the buffering capacity of the system and the displacement of hydrogen ions. Sodium nitrite may contribute to environmental stabilization by influencing the ionic balance and limiting degradation reactions, as reflected by the higher pH values in the variants containing nitrite (S_50–S_100, Table 1). The impact of reducing the sodium nitrite dose on the pH value of meat products has also been evaluated by other researchers. Aksu et al. [29], in a study on *Pastırma*, a traditional dry-cured meat product, reported no statistically significant differences in pH among samples with varying nitrite concentrations; however, the highest pH value was consistently observed in the nitrite-free variant.

### 4.2. Oxidative Stability

Furthermore, an increase in oxidation-reduction potential (ORP) was recorded across all treatment groups. The lowest ORP value was observed in the sample containing the full dose of sodium nitrite (S_100), while the highest was found in the nitrite-free sample (S_0). The ORP parameter reflects the oxidative-reductive balance within the meat matrix. A lower ORP indicates a more reducing environment, which is favorable for maintaining the stability of lipids and meat pigments. Samples supplemented with sodium nitrite exhibited lower ORP values, which can be attributed to the reduction of nitrite to nitrite under low-oxygen conditions, followed by further conversion to nitric oxide (NO). Nitric oxide is capable of scavenging free oxygen radicals, thereby mitigating oxidative degradation. In contrast, the absence of sodium nitrite in the S_0 variant resulted in the lack of such reactive, reducing intermediates, leading to a higher ORP and increased susceptibility to oxidative reactions. This observation was corroborated by thiobarbituric acid reactive substances (TBARS) analysis conducted after 30 days of storage. All samples exhibited increased TBARS values over time; however, the lowest levels were recorded in the S_100 variant, while the highest were noted in S_0. This is reflected in the TBARS values, which increased after 30 days of storage in each sample, with the lowest levels recorded in S_100 and the highest noted in S_0; however, variant S_75 showed similar effectiveness to S_100. Comparable results were obtained by Karwowska et al. [30], who examined cooked meat products formulated with 0, 50, 100, and 150 mg NaNO_2_ kg^−1^. Their data showed a clear inverse relationship between nitrite concentration and lipid oxidation: complete omission of nitrite increased TBARS from 0.43 to 3.14 mg kg^−1^ after 15 days of storage, whereas supplementation with 50 mg kg^−1^ produced TBARS levels comparable to those achieved with 100–150 mg kg^−1^. Subsequent studies by Guo et al. [31] confirmed that NaNO_2_ markedly slows TBARS accumulation relative to untreated controls, underscoring the intrinsic antioxidant function of nitrite. This antioxidant effect is particularly important from a health perspective, since lipid oxidation products such as malondialdehyde (MDA) are involved in cellular damage and inflammation after consumption. Therefore, maintaining a low level of lipid oxidation in meat products helps to reduce potential negative health effects.

TBARS quantifies malondialdehyde (MDA), a secondary oxidation product formed during the propagation phase of polyunsaturated-fatty-acid oxidation; thus, elevated TBARS values signify more advanced oxidative deterioration. The presence of sodium nitrate/nitrite inhibits the initiation and propagation of lipid-oxidation chain reactions because its reduction products, most notably nitric oxide (NO), can effectively neutralize peroxyl radicals [32]. Other authors have demonstrated that the use of a freeze-dried or fresh CFS of lactic acid bacteria can effectively reduce oxidative processes in foods, such as meat [8,33] or shrimp [34]. The addition of CFS led to a significant reduction in TBARS values, indicating a slower formation of secondary lipid oxidation products. This effect was particularly evident during refrigerated storage, where CFS acted synergistically with natural antioxidants and compounds with antimicrobial activity. Consequently, this contributed to an extended shelf life and improved sensory quality of the food.

As a result, oxidation processes are limited, which is confirmed by the low TBARS in S_100. In the S_75 sample, a similar effect was probably achieved due to synergy with the addition of CFS, which could introduce bioactive compounds with antioxidant activity. Similarly, the role of CFS seems to be important considering the profile of fatty acids. PUFA are most susceptible to oxidation. Their lower level in S_100 after storage may indicate that, despite protection against secondary oxidation (low TBARS), the initial decomposition of polyunsaturated fatty acids occurred. The high PUFA content in S_75 suggests that this variant better protected lipids from degradation, probably due to the synergy of sodium nitrite with the natural antioxidant from CFS. Variant S_0 contained less PUFA and more SFA, which may indicate greater degradation of fatty acids due to the lack of protection against oxidation. Similarly, in the study by Karwowska et al. [35], in dry-cured loins, it was found that the amount of PUFA decreases during storage, while samples with the addition of native and autoclaved mustard and acid whey (containing bioactive compounds from LAB) to sausages without nitrites were able to protect PUFA against oxidation comparable to nitrites.

The level of oxidation may also depend on the antioxidant profile of the meat matrix. In this study, the ABTS and RP methods were used to assess this issue. At the beginning (day 1), the highest ABTS activity was shown by samples S_100 and S_75. After 30 days, there was a decrease in activity in S_100 and S_75, and a slight increase in S_50 and S_0. However, the relationship between the variants remained unchanged: S_100 > S_75 > S_50 > S_0. The ABTS method measures the ability of a substance to neutralize free radicals (radical scavenging). High activity in S_100 and S_75 is associated with the presence of NO and potentially other reducing compounds generated from nitrites, which react quickly with radicals. The role of CFS in shaping the antioxidant effect of the meat matrix was also emphasized in the literature. In another study, it was shown that CFS from *L. casei* used in ground meat showed high ABTS activity and was rich in polyphenols/flavonoids, suggesting effective antioxidant protection of CFS in the meat model [36]. Also, a recent study by Ozturk and Sengun [7] showed that CFS from selected LAB strains showed high ABTS neutralization capacity. The decrease in activity after 30 days observed in this study may be due to the depletion of active antioxidants (nitrites, NO, natural phenols from CFS) during longer storage. Previous reports have repeatedly indicated that nitrites, NO, and natural antioxidant compounds are consumed over time during storage and thermal processing. For example, Morrissey and Tichivangana [37] suggest that NO–myoglobin complexes and iron chelates are depleted during storage, reducing the active pool of nitrite and NO, which translates into a decrease in antioxidant activity. In turn, a small increase in ABTS in S_0 and S_50 at day 30 of meat preserve storage may be the result of secondary products of protein or lipid decomposition, which secondarily show antioxidant activity but are less effective. According to Domínguez et al. [38], during the long-term storage of meat products, lipid and protein oxidation processes intensify, resulting in the formation of secondary oxidation products. The authors emphasize that some of these compounds formed as a result of the degradation of meat components, such as Maillard reaction products, oxidized peptides, or low-molecular-weight aldehydes, and may demonstrate the ability to scavenge free radicals, which translates into an increase in the values determined by ABTS methods. The observed increase in antiradical activity in ABTS tests in variants with reduced nitrite content (such as S_0 or S_50) may be the result of the accumulation of such secondary oxidation products.

At the beginning, the lowest RP was shown by variant S_100, and the highest was shown by S_50 and S_75. After 30 days, all variants showed similar, higher RP, except for S_0, which still had the lowest level. RP measures the ability of samples to transfer electrons (reduce Fe^3+^ to Fe^2+^). The initially lower RP in S_100 may be due to the presence of sodium nitrite in a stable form, not yet active as a reducing agent. After 30 days, as a result of storage, chemical changes may occur, which lead to the formation of secondary reducing compounds, which was manifested by an increase in reducing power. This increase may be the result of chemical changes during storage, leading to the release of active reducing compounds (e.g. amines, peptides, or lipid degradation products) or their conversion to more active forms [39,40].

### 4.3. Color and Texture

The applied technological treatment (introduction of 1% CFS and limitation of sodium nitrite) also had an effect on the color and texture of preserved meat. Sodium nitrite (through the formation of nitrosylmyoglobin) stabilizes the intense color of the meat and prevents the lightening of the product. Nitrite (or nitrate converted to nitrite) reacts with myoglobin, initially forming nitrosylmyoglobin, which, during heat treatment, transforms into a stable pink-red pigment called nitrosohemochrome, responsible for the intense pink color of cured meat [41,42]. Moreover, Wójciak et al. [42] demonstrated that the dose of sodium nitrite had a statistically significant effect on the lightness and yellowness of roasted beef. In S_0, where a significant increase in lightness was noted after 30 days, there was probably a partial denaturation of myoglobin and degradation of pigments due to the lack of a stabilizing factor (sodium nitrite), which results in greater light reflection and an increase in L*. The same mechanism is responsible for changes in redness in the nitrite-free variant (S_0), while the remaining variants with nitrite presented constant values. The red color of cured meat products is one of the important effects of nitrite in meat products. Nitric oxide (NO) is formed as a result of the reaction between sodium nitrite and the iron present in myoglobin and metmyoglobin, leading to the formation of nitrosylmyoglobin, which gives cured meat its characteristic red color [42]. As a result of oxidation and the denaturation of pigment proteins, a* decreases. In the case of the next color parameter, yellowness, its increase was observed with decreasing nitrogen dose. This was manifested by a shift in the color of the meat towards yellow-brown, typical of oxidized myoglobin and lipid degradation products. Since the highest b* values characterized the S_0 variant, it is possible that oxidation products accumulate in the product due to the lack of sufficient antioxidant protection. Additionally, the nitrite-free S_0 variant showed higher chromaticity, with a simultaneous lack of red stabilization (decrease in a*) and a higher color angle (H°), which confirms the dominance of yellow-brown tones (related to b*) as a result of dye oxidation and the lack of nitrosylation reaction. At the same time, the variants with nitrite retain lower H°, corresponding to the classic color of cured meat (pink-red), confirming the stabilizing, color-forming role of nitrites. The literature has shown that the lack of nitrites leads to the oxidation of MbO_2_ to methylmyoglobin (Met-Mb), which is responsible for the characteristic yellow-brown colors [43]. Classical color parameters did not provide a clear answer about the role of CFS addition in its features. Only Δ*E* indicated that a 25% reduction of nitrite (S_75) did not significantly affect color perception (Δ*E* < 3 means a color change not visible to the naked eye), which may have practical technological significance. The greatest color changes (S_0, Δ*E* > 8) result from the lack of formation of colored myoglobin complexes with NO and from the intensification of pigment oxidation processes.

The study showed that the use of CFS additive combined with a reduction in sodium nitrite content not only affects the coloring effect of meat, but also significantly influences the textural properties of canned pork. The presence of sodium nitrite contributed to the stabilization of the structure of the gelling protein network, probably by limiting their oxidative degradation, which helped to maintain the structural integrity of the meat. Sodium nitrite limits protein oxidation (smaller carbonyl groups, dityrosine), prevents changes in the secondary structure (from α to β), increases the available –SH form, and reduces surface hydrophobicity, which confirms the role of this additive in the stabilization of the protein gelling network [44,45]. This phenomenon is confirmed by, among others, Govari and Pexara [46], who showed that nitrates and nitrites, used as curing additives, protect meat proteins against oxidation, maintaining their structure and functionality by stabilizing heme iron, reacting with radicals and protecting SH groups. As the studies by Wang et al. [45] indicated, even at a low dose of nitrite (50 mg kg^−1^), there was significant protection of meat proteins against oxidation. The lack of sodium nitrite in the S_0 variant could lead to faster dehydration and the excessive stiffening of the structure as soon as the first day of storage, which is reflected in the highest recorded hardness. Similar observations were made by Bao and Ertbjerg [47] and by Yu et al. [48], who showed that, in conditions of increased oxidation, protein denaturation, the deterioration of water-binding capacity, and unfavorable textural changes (increased hardness, brittleness) occur. In this study, after 30 days of storage, a decrease in hardness was observed in all samples, which indicates the progressive degradation of the muscle gel. At the same time, chewiness and gumminess increased with time and with decreasing sodium nitrite content, reaching the highest values in samples without its addition (S_0). These phenomena may be the result of the formation of stiffer, less flexible protein networks in the absence of nitrite, as well as greater susceptibility to oxidative changes [39]. Xiao-Hui et al. [49] documented that the use of nitrates significantly affects the texture and elasticity of meat by modifying gelation, which, in turn, affects their water-binding capacity. Variants containing CFS, especially S_75, showed improved textural properties. It is possible that bioactive compounds present in CFS, such as peptides, organic acids, and phenolic metabolites, acted protectively on proteins, limiting their oxidation during heat treatment and storage. As shown by Humam et al. [50], selected CFS metabolites have antioxidant and protease-inhibiting properties, which promotes the preservation of gel strength and limits textural deterioration in heat-treated meat products. Similarly, Wang et al. [51] noted that the use of LAB and their metabolites improves the elasticity and cohesion of fermented meat, probably by limiting oxidation and modulating protein interactions.

### 4.4. Microbiology

Although this study did not analyze the presence of spore-forming anaerobic bacteria, including *Clostridium* spp., it should be clearly stated that the potential risk of their presence in canned meats cannot be ruled out, especially with reduced nitrite addition, which plays a significant role in inhibiting the growth of these microorganisms. Importantly, this work did not include microbiological challenge tests or any direct assessment of *Clostridium* growth, which highlights the need for additional microbiological assessment of canned meats under reduced nitrite conditions in future studies. However, epidemiological data indicate that *Clostridium botulinum* food contamination remains low and stable, and most cases of foodborne botulism are associated with improper storage and handling of food products at home [52]. Recent literature also highlights that nitrite reduction may increase the risk of botulism under certain conditions, particularly in products with higher pH and water activity, emphasizing the need for careful formulation and additional safety testing [53,54,55]. Therefore, the study focused on the analysis of aerobic microflora, which plays a key role in the spoilage of canned meat products after opening and during subsequent storage. The microbiological analysis showed that reducing the dose of sodium nitrite in the canned pork had no significant effect on the total number of aerobic bacteria, lactic acid bacteria, or coliform bacteria (*p* > 0.05), which suggests that reducing this preservative does not worsen the overall microbiological stability of the product. Regardless of the sodium nitrite dose used (S_0–S_100), the average number of coliform bacteria, aerobic microorganisms, and lactic acid bacteria remained at a comparable level. However, the qualitative analysis of the microbiota showed variability in the dominant bacterial species, depending on the level of sodium nitrite addition. In samples without sodium nitrite addition (S_0), the most frequently isolated were *Enterobacter cloacae* (37%) and *Serratia liquefaciens* (34%), while, at the dose of 50 mg kg^−1^, *S. liquefaciens* dominated (46%). In the 75 mg kg^−1^ variant, *Pantoea agglomerans* (31%) was also predominant. It is worth noting that the S_0 sample without nitrite contained a much greater diversity of genera and species of identified microorganisms than the samples with higher nitrite additions. This may indicate that nitrites have a preservative effect and that only a few groups of microorganisms are resistant to them. Therefore, despite the lack of quantitative differences, we see qualitative differences: a reduction in microbial diversity with increasing nitrite addition. The changing composition of the microbiota may be important for food safety, because some of the detected species, such as *E. cloacae* or *S. liquefaciens*, are opportunistic pathogens capable of causing infections in immunocompromised individuals. Therefore, despite the lack of differences in the total number of microorganisms, changes in the structure of the microbiota may be of significance for public health.

## 5. Conclusions

The study of the quality of preserved pork with the addition of 1% freeze-dried cell-free supernatant (CFS) from *Lacticaseibacillus paracasei* B1, combined with reduced sodium nitrite levels, has important implications for consumer health. The results indicate that partial reduction of sodium nitrite in pork canned meats fortified with 1% CFS from *Lacticaseibacillus paracasei* B1 can be implemented without substantially altering the physicochemical characteristics of the product. Variant S_75, containing 75% of the standard dose of nitrite, showed the best balance between color stability, textural integrity, and oxidative stability during storage, suggesting that it is a viable alternative to nitrite reduction. However, complete omission of sodium nitrite (S_0) negatively affected sensory attributes such as color and texture, and led to increased oxidative degradation. These results indicate that CFS can help to reduce nitrites by up to 25% without compromising key quality attributes, providing a practical strategy for improving the health profile of processed meat products while maintaining product quality.

## Figures and Tables

**Table 1:** Physicochemical parameters of canned pork meat with 1% CFS and reduced nitrite amount after production (day 1) and storage (4 °C, day 30) (means ± standard error).

Storage [Day]	S_100	S_75	S_50	S_0
	water activity			
1	0.985 ± 0.002 *^aA^*	0.980 ± 0.002 *^aA^*	0.985 ± 0.004 *^aA^*	0.987 ± 0.04 *^aA^*
30	0.967 ± 0.02 *^aB^*	0.968 ± 0.001 *^aB^*	0.964 ± 0.001 *^aB^*	0.958 ± 0.002 *^bB^*
	pH			
1	5.968 ± 0.049 *^aA^*	5.870 ± 0.021 *^bA^*	5.835 ± 0.005 *^cA^*	5.782 ± 0.013 *^cA^*
30	6.165 ± 0.049 *^aB^*	6.063 ± 0.072 *^abB^*	6.070 ± 0.010 *^abB^*	6.057 ± 0.006 *^bB^*
	oxidation-reduction potential			
1	342.933 ± 7.574 *^aA^*	339.633 ± 1.528 *^aA^*	340.400 ± 2.265 *^aA^*	352.167 ± 5.543 *^aA^*
30	393.100 ± 3.253 *^aB^*	408.457 ± 3.669 *^bcB^*	400.500 ± 5.027 *^abB^*	414.800 ± 6.843 *^cB^*

[S_100—variant with a sodium nitrite dose of 100 mg/kg; S_75—variant with a sodium nitrite dose of 75 mg kg^−1^; S_50—variant with a sodium nitrite dose of 50 mg kg^−1^; S_0—nitrite-free variant. *^a–c^*—different lowercase letters indicate significant differences between variants within the same day; *^A,B^*—different uppercase letters indicate significant differences between days within the same variant (*p* < 0.05)].

**Table 2:** Main fractions and fatty acid profile of canned pork meat with 1% CFS and reduced nitrite amount after storage (30 days) (means ± standard error).

Fatty Acid	S_100	S_75	S_50	S_0
C6:0	0.08 ± 0.10 *^a^*	0.09 ± 0.005 *^a^*	0.09 ± 0.002 *^a^*	0.08 ± 0.002 *^a^*
C8:0	0.08 ± 0.05 *^a^*	0.08 ± 0.002 *^a^*	0.08 ± 0.010 *^a^*	0.90 ± 0.010 *^b^*
C10:0	0.13 ± 0.02 *^a^*	0.30 ± 0.005 *^b^*	0.14 ± 0.05 *^c^*	0.11 ± 0.005 *^d^*
C12:0	0.11 ± 0.010 *^a^*	0.11 ± 0.005 *^a^*	0.11 ± 0.05 *^a^*	0.10 ± 0.002 *^a^*
C14:0	1.69 ± 0.005 *^a^*	1.59 ± 0.010 *^b^*	1.66 ± 0.008 *^c^*	1.61 ± 0.005 *^d^*
C14:1n5	0.02 ± 0.005 *^a^*	0.02 ± 0.005 *^a^*	0.02 ± 0.002 *^a^*	0.02 ± 0.005 *^a^*
C15:0	0.048 ± 0.008 *^a^*	0.047 ± 0.006 *^a^*	0.05 ± 0.030 *^a^*	0.05 ± 0.005 *^a^*
C16:0	29.56 ± 0.002 *^a^*	28.53 ± 0.002 *^b^*	29.19 ± 0.005 *^c^*	29.11 ± 0.002 *^d^*
C16:1n7	2.98 ± 0.030 *^a^*	3.06 ± 0.010 *^b^*	3.06 ± 0.010 *^b^*	2.88 ± 0.002 *^c^*
C17:0	0.23 ± 0.005 *^a^*	0.24 ± 0.005 *^ab^*	0.25 ± 0.005 *^b^*	0.23 ± 0.10 *^a^*
C17:1n7	0.17 ± 0.002 *^a^*	0.20 ± 0.10 *^b^*	0.20 ± 0.005 *^b^*	0.17 ± 0.005 *^a^*
C18:0	16.19 ± 0.010 *^a^*	15.24 ± 0.002 *^b^*	15.50 ± 0.010 *^c^*	16.39 ± 0.005 *^d^*
C18:1n9c + C18:1n9t	39.14 ± 0.010 *^a^*	40.40 ± 0.002 *^b^*	36.64 ± 0.005 *^c^*	39.92 ± 0.005 *^d^*
C18:2n6c + C18:2n6t	2.1 ± 0.005 *^a^*	2.81 ± 0.030 *^b^*	2.08 ± 0.005 *^a^*	2.39 ± 0.002 *^c^*
C18:3n3 (ALPHA)	0.04 ± 0.002 *^a^*	0.09 ± 0.002 *^b^*	0.04 ± 0.002 *^a^*	0.07 ± 0.005 *^c^*
C20:0	0.21 ± 0.010 *^a^*	0.20 ± 0.030 *^a^*	0.20 ± 0.010 *^a^*	0.22 ± 0.005 *^a^*
C20:1n15	0.77 ± 0.005 *^a^*	0.77 ± 0.005 *^a^*	0.77 ± 0.005 *^a^*	0.78 ± 0.002 *^a^*
C20:1n9	0.12 ± 0.010 *^a^*	0.15 ± 0.002 *^b^*	0.11 ± 0.010 *^a^*	0.13 ± 0.010 *^ab^*
C20:3n3	0.03 ± 0.005 *^a^*	0.03 ± 0.005 *^a^*	0.02 ± 0.008 *^a^*	0.05 ± 0.005 *^b^*
C22:0	0.07 ± 0.010 *^a^*	0.07 ± 0.005 *^a^*	0.05 ± 0.005 *^b^*	0.06 ± 0.002 *^a^*
C24:1n9	0.42 ± 0.005 *^a^*	0.33 ± 0.010 *^b^*	0.37 ± 0.002 *^c^*	0.32 ± 0.00 ^b^
	SUMMARY			
SFA	48.48 ± 0.027 *^a^*	46.35 ± 0.287 *^b^*	47.40 ± 0.027 *^c^*	48.50 ± 0.287 *^a^*
MUFA	43.62 ± 0.046 *^a^*	44.93 ± 0.046 *^b^*	44.17 ± 0.037 *^c^*	44.22 ± 0.046 *^c^*
PUFA	2.08 ± 0.035 *^a^*	2.97 ± 0.033 *^b^*	2.24 ± 0.013 *^c^*	2.55 ± 0.033 *^d^*
OMEGA3	0.07 ± 0.08 *^a^*	0.14 ± 0.001 *^b^*	0.07 ± 0.009 *^a^*	0.12 ± 0.001 *^c^*
OMEGA6	2.10 ± 0.042 *^a^*	2.83 ± 0.040 *^b^*	2.17 ± 0.042 *^a^*	2.43 ± 0.040 *^c^*
OMEGA9	39.68 ± 0.092 *^a^*	40.88 ± 0.024 *^b^*	40.12 ± 0.052 *^c^*	40.37 ± 0.024 *^d^*

[S_100—variant with a sodium nitrite dose of 100 mg kg^−1^; S_75—variant with a sodium nitrite dose of 75 mg kg^−1^; S_50—variant with a sodium nitrite dose of 50 mg kg^−1^; S_0—nitrite-free variant. *^a–d^*—different lowercase letters indicate significant differences between variants within the same day].

**Table 3:** Antioxidant potential of meat extract obtained from canned meat with reduced sodium nitrite content and 1% addition of CFS lyophilizate after production (day 1) and storage (4 °C; day 30) (means ± standard error).

Storage [Day]	S_100	S_75	S_50	S_0
	ABTS [%]			
1	77.08 ± 1.19 *^cA^*	73.23 ± 4.52 *^cA^*	57.52 ± 3.39 *^bA^*	47.13 ± 2.73 *^aA^*
30	72.71 ± 3.52 *^cB^*	61.10 ± 2.09 *^bA^*	58.60 ± 1.54 *^bB^*	54.64 ± 1.42 *^aB^*
	RP [A_700_]			
1	0.16 ± 0.07 *^cA^*	1.60 ± 0.01 *^bA^*	1.62 ± 0.06 *^bA^*	1.40 ± 0.05 *^aA^*
30	1.66 ± 0.02 *^bB^*	1.68 ± 0.03 *^bB^*	1.71 ± 0.02 *^bB^*	1.54 ± 0.01 *^aB^*

[ABTS—antioxidant activity again ABTS radicals; RP—reducing power. S_100—variant with a sodium nitrite dose of 100 mg kg^−1^; S_75—variant with a sodium nitrite dose of 75 mg kg^−1^; S_50—variant with a sodium nitrite dose of 50 mg kg^−1^; S_0—nitrite-free variant. *^a–c^*—different lowercase letters indicate significant differences between variants within the same day; *^A,B^*—different uppercase letters indicate significant differences between days within the same variant (*p* < 0.05)].

**Table 4:** Color parameters (CIE L*a*b*) of canned pork meat with 1% CFS and reduced nitrite amount after production (day 1) and storage (4 °C, day 30) (means ± standard error).

Storage [Day]	S_100	S_75	S_50	S_0
	lightness (L*)			
1	66.09 ± 1.70 *^aA^*	66.09 ± 1.70 *^aA^*	67.59 ± 2.54 *^aA^*	67.31 ± 2.70 *^aA^*
30	65.21 ± 1.31 *^aA^*	65.21 ± 1.31 *^aA^*	67.43 ± 1.58 *^bA^*	69.99 ± 1.56 *^cB^*
	redness (a*)			
1	9.15 ± 0.82 *^aA^*	9.15 ± 0.82 *^aA^*	8.07 ± 0.45 *^cA^*	3.61 ± 0.52 *^dA^*
30	9.34 ± 0.74 *^aA^*	9.34 ± 0.74 *^aA^*	8.18 ± 0.69 *^bA^*	5.11 ± 0.61 *^cB^*
	yellowness (b*)			
1	9.23 ± 0.60 *^aA^*	9.23 ± 0.60 *^aA^*	12.60 ± 0.49 *^bA^*	14.69 ± 0.96 *^cA^*
30	9.90 ± 0.87 *^aA^*	9.90 ± 0.87 *^aA^*	12.83 ± 0.50 *^bA^*	15.52 ± 0.31 *^cA^*
	chroma (C*)			
1	13.01 ± 1.44 *^aA^*	13.01 ± 1.44 *^aA^*	14.96 ± 1.42 *^bA^*	15.15 ± 1.71 *^bA^*
30	13.67 ± 0.91 *^aA^*	13.67 ± 0.91 *^aA^*	15.24 ± 0.41 *^bA^*	16.35 ± 0.29 *^cA^*
	hue angle (*H*°)			
1	45.30 ± 1.44 *^aA^*	45.30 ± 1.44 *^aA^*	57.43 ± 1.42 *^cA^*	76.20 ± 1.71 *^dA^*
30	46.95 ± 2.47 *^aA^*	46.95 ± 2.47 *^aA^*	57.50 ± 2.78 *^cA^*	71.86 ± 2.08 *^dB^*

[S_100—variant with a sodium nitrite dose of 100 mg kg^−1^; S_75—variant with a sodium nitrite dose of 75 mg kg^−1^; S_50—variant with a sodium nitrite dose of 50 mg kg^−1^; S_0—nitrite-free variant. *^a–c^*—different lowercase letters indicate significant differences between variants within the same day; *^A,B^*—different uppercase letters indicate significant differences between days within the same variant (*p* < 0.05)].

**Table 5:** Texture profile analysis (TPA) of canned meat with reduced sodium nitrite content and 1% addition of CFS lyophilizate after production (day 1) and storage (4 °C; day 30) (means ± standard error).

Storage [Day]	S_100	S_75	S_50	S_0
	Hardness [g]			
1	2053.44 ± 26.73 *^aA^*	2269.14 ± 55.08 *^bA^*	2833.60 ± 51.42 *^cA^*	2917.84 ± 58.36 *^dA^*
30	1656.90 ± 47.43 *^aB^*	1552.31 ± 47.43 *^abB^*	1727.12 ± 93.30 *^acB^*	2296.78 ± 28.21 *^dB^*
	Cohesion [-]			
1	0.349 ± 0.02 *^aA^*	0.332 ± 0.03 *^aA^*	0.351 ± 0.01 *^aA^*	0.270 ± 0.03 *^bA^*
30	0.586 ± 0.01 *^aB^*	0.657 ± 0.03 *^aB^*	0.653 ± 0.06 *^aB^*	0.582 ± 0.07 *^aB^*
	Springiness [%]			
1	70.695 ± 0.61 *^aA^*	68.04 ± 0.96 *^bA^*	62.904 ± 1.49 *^cA^*	76.230 ± 2.98 *^dA^*
30	85.313 ± 0.35 *^aB^*	84.076 ± 0.87 *^abB^*	82.918 ± 0.48 *^bB^*	80.116 ± 0.77 *^cA^*
	Gumminess			
1	718.43 ± 4.41 *^aA^*	760.37 ± 3.07 *^bA^*	808.77 ± 2.02 *^cA^*	868.23 ± 14.36 *^dA^*
30	970.218 ± 10.71 *^aB^*	1007.521 ± 7.67 *^bB^*	1234.153 ± 11.13 *^cB^*	1413.439 ± 19.18 *^dB^*
	Chewiness			
1	503.29 ± 7.24 *^aA^*	512.16 ± 8.64 *^aA^*	537.23 ± 6.56 *^bA^*	636.695 ± 7.43 *^cA^*
30	829.627 ± 4.78 *^aB^*	859.012 ± 3.65 *^aB^*	1008.642 ± 7.11 *^bB^*	922.153 ± 2.85 *^cB^*

[S_100—variant with a sodium nitrite dose of 100 mg kg^−1^; S_75—variant with a sodium nitrite dose of 75 mg kg^−1^; S_50—variant with a sodium nitrite dose of 50 mg kg^−1^; S_0—nitrite-free variant. *^a–d^*—different lowercase letters indicate significant differences between variants within the same day; *^A,B^*—different uppercase letters indicate significant differences between days within the same variant (*p* < 0.05)].

**Table 6:** Results of microbiological analysis of canned meat product (day 30) (means ± standard error).

Microbiological Criterium	S_100	S_75	S_50	S_0
Coliforms bacteria	1.56 ± 0.09 *^a^*	1.63 ± 0.13 *^a^*	1.65 ± 0.12 *^a^*	1.68 ± 0.15 *^a^*
Total viable count	1.70 ± 0.06 *^a^*	1.76 ± 0.09 *^a^*	1.68 ± 0.20 *^a^*	1.58 ± 0.11 *^a^*
Lactic acid bacteria	1.68 ± 0.15 *^a^*	1.65 ± 0.17 *^a^*	1.52 ± 0.29 *^a^*	1.56 ± 0.18 *^a^*

[S_100—variant with a sodium nitrite dose of 100 mg kg^−1^; S_75—variant with a sodium nitrite dose of 75 mg kg^−1^; S_50—variant with a sodium nitrite dose of 50 mg kg^−1^; S_0—nitrite-free variant. *^a^*—lowercase letters indicate significant differences between variants within the same day].

## Data Availability

The data presented in this study will be made available on request.
